# Simultaneous radical cystectomy and nephroureterectomy in the treatment of panurothelial carcinoma: a systematic review and single-arm meta-analysis

**DOI:** 10.3389/fonc.2023.1233125

**Published:** 2023-09-25

**Authors:** Yang Liu, Huimin Zhang, Zhi Wen, Yu Jiang, Jing Huang, Chongjian Wang, Caixia Chen, Jiahao Wang, Erhao Bao, Xuesong Yang

**Affiliations:** ^1^ Department of Urology, Affiliated Hospital of North Sichuan Medical College, Nanchong, China; ^2^ Department of Urology, Chengdu Xinhua Hospital Affiliated to North Sichuan Medical College, Chengdu, China; ^3^ Department of Radiology, Affiliated Hospital of North Sichuan Medical College, Nanchong, China

**Keywords:** radical cystectomy, nephroureterectomy, panurothelial carcinoma, UTUC, meta-analysis

## Abstract

**Background:**

Panurothelial carcinoma is a rare and aggressive malignancy that requires effective treatment strategies to enhance patient outcomes.

**Methods:**

We conducted a systematic search of English publications in databases including PubMed, Embase, Cochrane Library, and Web of Science up to May 2023. The quality of the literature was assessed using the Newcastle-Ottawa Scale (NOS) and the Methodological Quality and Synthesis of Case Series and Case Reports tool. Data statistics and analysis were performed using Stata 15.1 software (StataSE, USA).

**Results:**

Six studies involving 339 patients were included in the analysis. Meta-analysis revealed that Simultaneous Radical Cystectomy and Nephroureterectomy had 2-year and 5-year overall survival rates of 68% (95% CI 60%-76%, I^2^ = 12.4%, P < 0.001) and 44% (95% CI 36%-53%, I^2 ^= 0, P < 0.001), respectively. The 2-year and 5-year progression-free survival rates were 91% (95% CI 86%-95%, I^2 ^= 95%, P < 0.001) and 65% (95% CI 58%-73%, I^2 ^= 91.5%, P < 0.001), respectively. The 2-year and 5-year cancer-specific survival rates were 73% (95% CI 66%-81%, I^2 ^= 16.7%, P < 0.001) and 57% (95% CI 49%-66%, I^2 ^= 0, P < 0.001), respectively. Additionally, the incidence of minor complications was 19% (95% CI 15%-23%, P < 0.01), major complications was 49% (95% CI 34%-63%, P < 0.01), and the intraoperative blood transfusion rate was 53% (95% CI 44%-61%, P < 0.01).

**Conclusions:**

Simultaneous radical cystectomy and nephroureterectomy represent feasible approaches for the treatment of Panurothelial carcinoma. Nonetheless, a comprehensive assessment of the surgical risks and benefits is imperative, and larger-scale prospective cohort studies are required to validate therapeutic efficacy.

**Systematic review registration:**

https://www.crd.york.ac.uk/PROSPERO, identifier CRD42023426401.

## Introduction

Urothelial cell carcinoma (UCC) is emerging as the fourth most prevalent cancer in men, with 90%-95% of cases being bladder urothelial cancer and the majority of the remaining cases being upper tract urothelial carcinoma (UTUC) (1). The overall incidence rate of UTUC stands at 1.1 cases per 100,000 individuals, with a lower rate of 0.89 cases per 100,000 individuals for advanced-stage UTUC. Notably, in patients with metastatic UTUC, the overall one-year survival rate reaches 60.3% (2). Panurothelial carcinoma (Panuc) is a rare yet highly invasive malignancy (3). It represents the simultaneous occurrence of bladder urothelial cancer and UTUC (4, 5), accounting for approximately 11% of UTUC cases (6). Radical cystectomy (RC) is the established gold standard surgical procedure for treating invasive bladder urothelial cancer, while radical nephroureterectomy is the preferred treatment for invasive UTUC (7, 8). However, due to the rarity of Panurothelial carcinoma and the lack of original studies, there is currently no consensus on its optimal treatment strategy.

Previous small-scale case studies have reported on simultaneous radical cystectomy and nephroureterectomy (RCUN), suggesting that RCUN may reduce metastasis and disease recurrence. However, the safety and efficacy of RCUN in the treatment of Panuc patients remain unclear due to the limited sample sizes of these studies (9, 10), Recently, large-scale studies by Subiela et al. (11) and Britton et al. (12) have reported on the treatment of Panuc with RCUN. The study by Zein et al. (13) provided a comprehensive overview of perioperative outcomes in patients undergoing RCUN treatment for Panurothelial carcinoma. However, their included literature comprised numerous case reports and lacked statistical analyses of postoperative tumor survival data.

To better evaluate the effectiveness of RCUN in the treatment of Panuc, we conducted a systematic review and a single-arm meta-analysis. The aim of this study was to provide a comprehensive and scientifically grounded basis for the treatment of Panuc, offering valuable insights for clinical decision-making.

## Method

We conducted the present meta-analysis in accordance with the guidelines set forth by the Preferred Reporting Items for Systematic Reviews and Meta-Analyses (PRISMA) statement (14) Furthermore, we have registered the study in PROSPERO (CRD42023426401) to ensure transparency and adherence to best practices in systematic reviews and meta-analyses.

### Literature search strategy

We conducted a comprehensive search of the Embase, Web of Science, and Cochrane Library databases up until May 2023 to identify studies that met the inclusion criteria. Our search strategy involved combining relevant terms related to the intervention and the patient population: ((Radical Cystectomy OR Radical Cystectomies OR Radical Cystectom*) AND (Nephroureterectomy OR Nephroureterectomies OR Nephroureterectom*) AND (Upper tract urothelial carcinoma OR UTUC OR panurothelial carcinoma OR PanUC)). To ensure the comprehensiveness of our search, we also manually reviewed the references of relevant articles. Only studies reported in English were included.

### Study selection

The inclusion criteria were defined using the PICOS approach. The eligible studies needed to meet the following criteria: (1) patients diagnosed with Panuc, (2) patients who underwent simultaneous treatment with both RC and RCUN, (3) one or more of the following outcomes: perioperative, oncologic, or survival outcomes, and (4) study designs including cohort studies, single-arm studies, or randomized controlled trials (RCTs). Studies were excluded if they met any of the following criteria: (1) unavailable data for analysis, (2) comments, reviews, case reports, abstracts, or editorials, (3) duplicate publications.

### Data collection

The data extraction process was carried out independently by two reviewers. The extracted data included the following information: (1) general information such as the first author, publication year, and country; (2) population characteristics including the number of patients, age, body mass index (BMI), median follow-up time, pathological stage, and outcomes; (3) perioperative outcomes such as operative time, estimated blood loss, transfusion rates, and length of stay; (4) assessment of minor complications (defined as Clavien grade 1-2) and major complications (defined as Clavien grade ≥3); (5) survival data including overall survival (OS), recurrence-free survival (RFS), and cancer-specific survival (CSS). Any discrepancies that arose during the data extraction process were resolved through consensus or consultation with a third reviewer. Only studies reported in English were included in the reference list.

### Bias risk assessment

The quality of the included publications was assessed using the Newcastle-Ottawa Scale (NOS) (https://www.ohri.ca//programs/clinical_epidemiology/oxford.asp) (15). The NOS was used to evaluate the case selection, comparability, and outcome reporting of each study. Two authors independently conducted the quality assessment, and any disagreements were resolved through discussion and consensus among all authors. For case series and case reports, the methodological quality and synthesis were evaluated using the “Methodological quality and synthesis of case series and case reports” tool (16).

### Statistical analysis

We utilized STATA 15.1 software (StataSE, USA) to perform a meta-analysis combining overall survival (OS), recurrence-free survival (RFS), cancer-specific survival (CSS), and perioperative outcomes in patients who underwent simultaneous radical cystectomy and nephroureterectomy (RCUN) for Panuc. KM curve data was extracted and estimated using Digitizer 4.1 and Adobe Photoshop (17). All pooled results were presented with 95% confidence intervals (CIs). Inter-study heterogeneity was assessed using the I^2^ statistic and the Cochran Q test (18). For studies with high heterogeneity (I^2^ ≥ 50%), a random-effects model was employed, while studies with low heterogeneity (I^2^ ≤ 50%) were analyzed using a fixed-effects model. Statistical significance was considered at a P-value < 0.05. Sensitivity analysis was conducted by excluding each study individually to assess the robustness of our findings. However, due to the limited number of studies (three or fewer), sensitivity analysis could not be performed.As the number of included studies was ten or fewer, the power of the tests for publication bias assessment was inadequate (19, 20). Hence, we were unable to conduct a thorough evaluation of publication bias.

## Results

### Baseline characteristics

**Figure 1:**
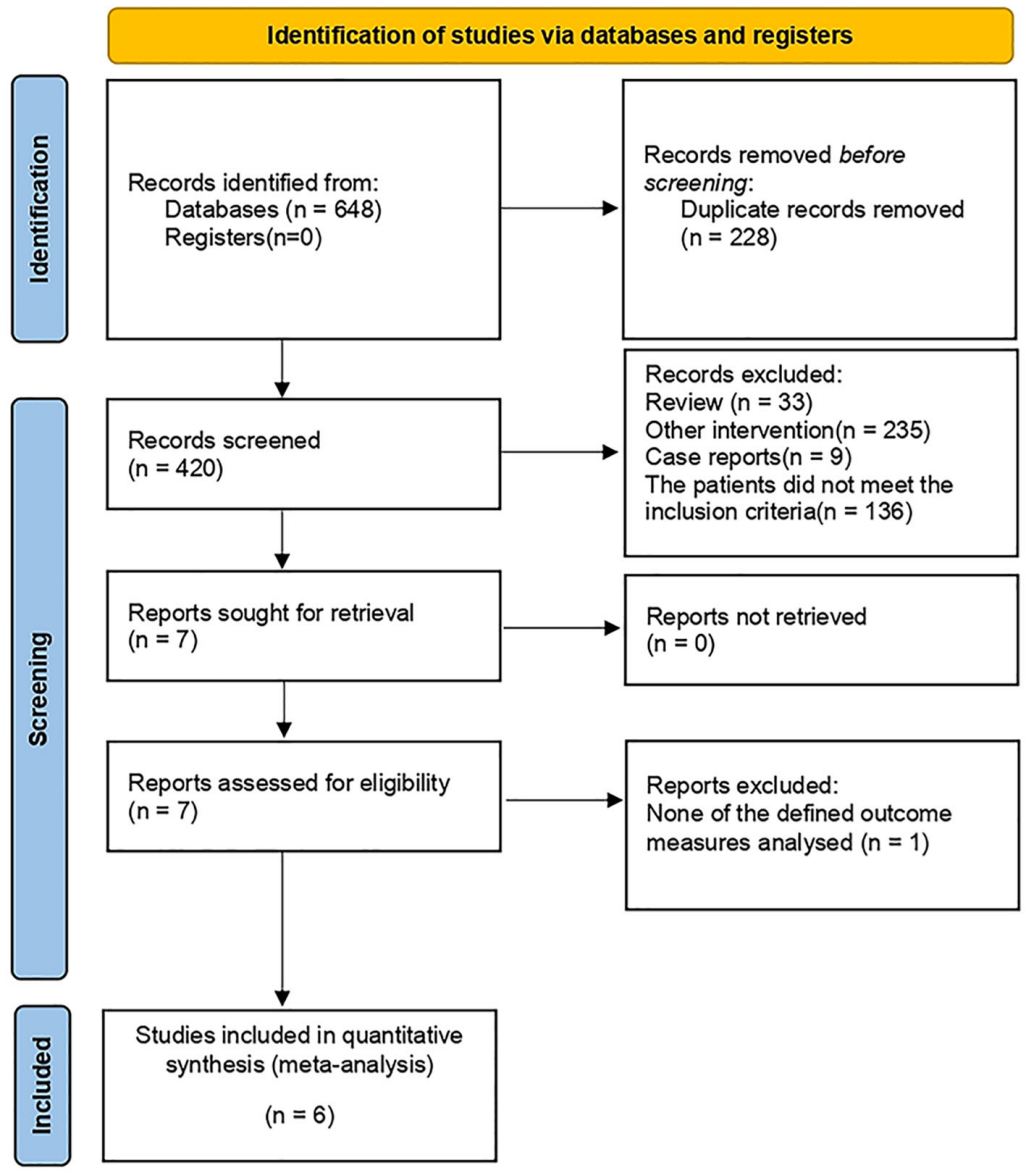
Literature screening flowchart.

A total of 648 studies were retrieved. After initial screening, we identified 7 potentially eligible studies, of which 6 studies met the inclusion criteria and were included in our analysis (9–12, 21, 22) (**Figure 1**).

The included studies originated from the USA, China, and Spain. The sample sizes ranged from 8 to 170 patients, totaling 339 patients who underwent simultaneous radical cystectomy and nephroureterectomy for Panurothelial Carcinoma. Open surgery accounted for 64% of the total cases, while minimally invasive procedures constituted 36% of the total cohort. **Table 1** summarizes the age, BMI, gender, date of follow-up, and other baseline information of the included patients. **Table 2** provides an overview of the tumor stage, history of previous abdominal surgery, history of previous chemotherapy, and other relevant data for the included patients. **Table 3** presents the quality evaluation of the case series.

**Table 1:** Characteristics of studies and patient populations.

Study	Country	No.of patient	Gender(male/femal)	BMI	Age(years)	Charlson comorbidity index	ASA score	Median follow-up time	NOS
Barros2008	USA	8	7 \1	25.8(9.6)	76.5(65-79)	NA	3.25(0.6)	9(1-45)	–
Britton2023	USA	39	32 \7	28.1(2.8)	72(65-76))	1.9(1.1)	NA	6.3(1.9-11.1)	7
Carpinito2022	USA	27	22 \5	28(4.4)	71(66-75)	4(3.5-7)	2.1(0.36)	36(11-52)	–
Kanabur2022	USA	190	145 \45	NA	69(55-83)	NA	NA	NA	–
Ou2010	China	8	5 \3	24.6(3.7)	66.9(6.6)	NA	2.8(0.3)	28.1(2-54)	–
Subiela2023	Spain	67	57 \ 10	26.6(23-29.1)	64(56-71)	5(4-5)	2.8(0.6)	38(17-88)	–

**Table 2:** Tumor staging and pathological data of patient populations.

Study	No. of Pelvic Lymph Nodes Dissected, n(%)	Upper Tract Involvement,n	Neoadjuvant Chemotherapy	Previous Surgery,n(%)	RC Pathology	NU Pathology	operative approach,n
Barros2008	7(87.5%)	renal pelvis and ureter:(n=2) ureter:(n=3) renal pelvis:(n=1)	75%	7(87.5%)	pT1G3:1; pT4G3:1; pT2 G2:3; pT0:1; pT3 G2:1; pTis:1	Ptis:2; pT0:2; pT1:1;pTa:1; pT2:1; pT3:1	Laparoscopy(n=7), Robotic(n=1)
Britton2023	31(79)	NA	10.30%	NA	29 (74.4%)<pT2; 6 (15.4%)pT2; 4 (10.3%)>pT2; 37 (94.9%)N0/X, 2 (5.1%) N+;	Tis7 (17.5%); Ta12 (30.8%); T0 1 (2.5%); T1 10 (25.0%); T2 8 (20.0%)T3 1 (2.5%);	Laparoscopy(n=4), Robotic(n=1), Open(n=34)
Carpinito2022	NA	NA	8 (30%)	5(18.5%)	cTa/Tis 13 (48%) cT1 4 (15%) cT2 + 10 (37%)	cTa/Tis 11 (41%) cT1 1 (3.7%) cT2 + 1 (3.7%)	Laparoscopy(n=9), Robotic(n=5), Open(n=13)
Kanabur2022	NA	NA	NA	NA	NA	NA	Open(n=125), Laparoscopy(n=65)
Ou2010	8(100)	NA	NA	NA	T1, high 3; T1, low 1; CIS, high 1; CIS, low1	T1 3; Ta 2; T2a 1;	Robotic(n=8)
Subiela2023	67(100)	Renal pelvis 23 (34.3%) Proximal ureter 13 (19.4%) Distal ureter 27 (49.3%) Pelvis and ureter 4 (6%)	33 (49.3%)	19(28.4%)	G1/G2 37 (55.3) G3 30 (44.7)	NMI disease (Tis, Ta, T1) 41 (61.2%); T2 6 (8%); T3-T4 20 (29.8%)	Open(n=45), Laparoscopy(n=22)

NA, Not Available.

**Table 3:** Quality assessment of case series.

Description	Selection	Ascertainment		Causality				Reporting
	1. Does the patient(s) represent(s) the whole experience of the investigator (centre) or is the selection method unclear to the extent that other patients with similar presentation may not have been reported	2. Was the exposure adequately ascertained?	3. Was the outcome adequately ascertained?	4. Were other alternative causes that may explain the observation ruled out?	5. Was there a challenge/rechallenge phenomenon?	6. Was there a dose– response effect?	7. Was follow-up long enough for outcomes to occur?	8. Is the case(s) described with sufficient details to allow other investigators to replicate the research or to a llow practitioners make inferences related to their own practice?
Barros2008	U	Y	Y	Y	Y	N	Y	Y
Carpinito2022	U	Y	Y	Y	Y	N	Y	Y
Ou2010	U	Y	Y	Y	Y	N	Y	Y
Subiela2023	U	Y	Y	Y	Y	N	Y	Y
Y, yes; U, unclear; N, no.								

### Outcome analysis

#### Perioperative outcomes

**Figure 2:**
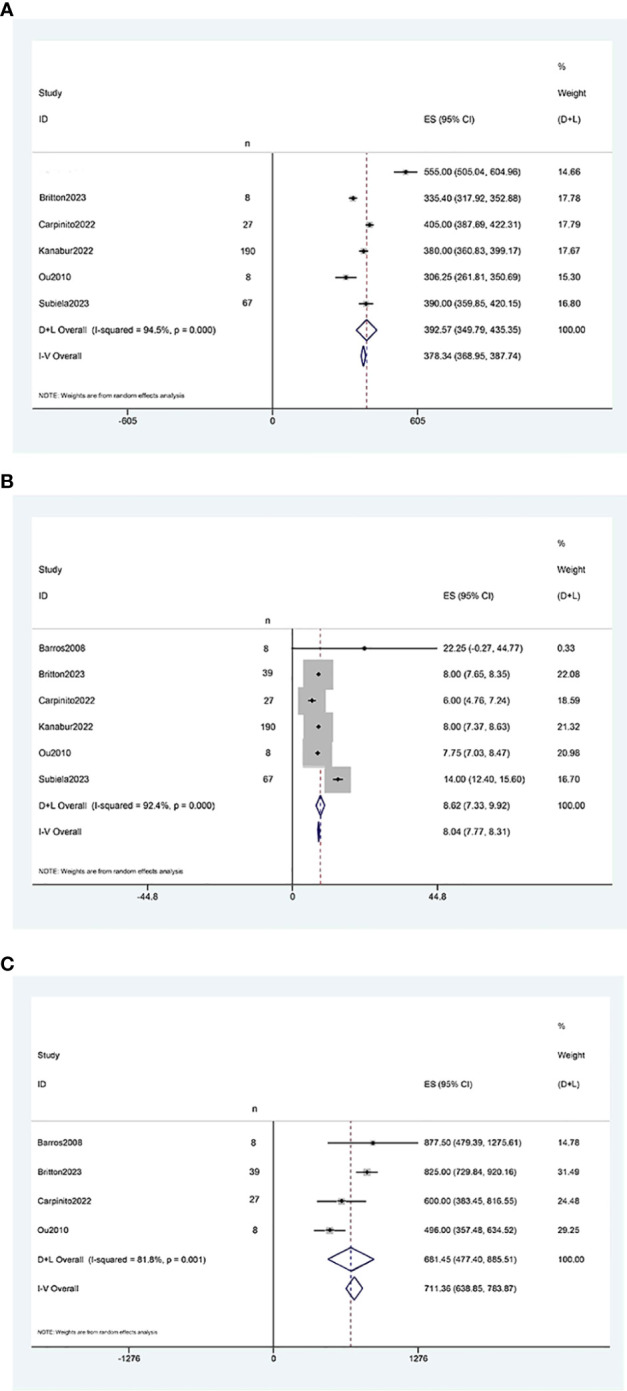
Forest plots of Perioperative outcome: **(A)** operation time was 378.34 minutes (95% CI 368.95, 387.74); **(B)** length of hospital stay was 8.04 days (95% CI 7.77, 8.31); **(C)** blood loss was 711.36 ml (95% CI 638.85, 783.87).

**Figure 3:**
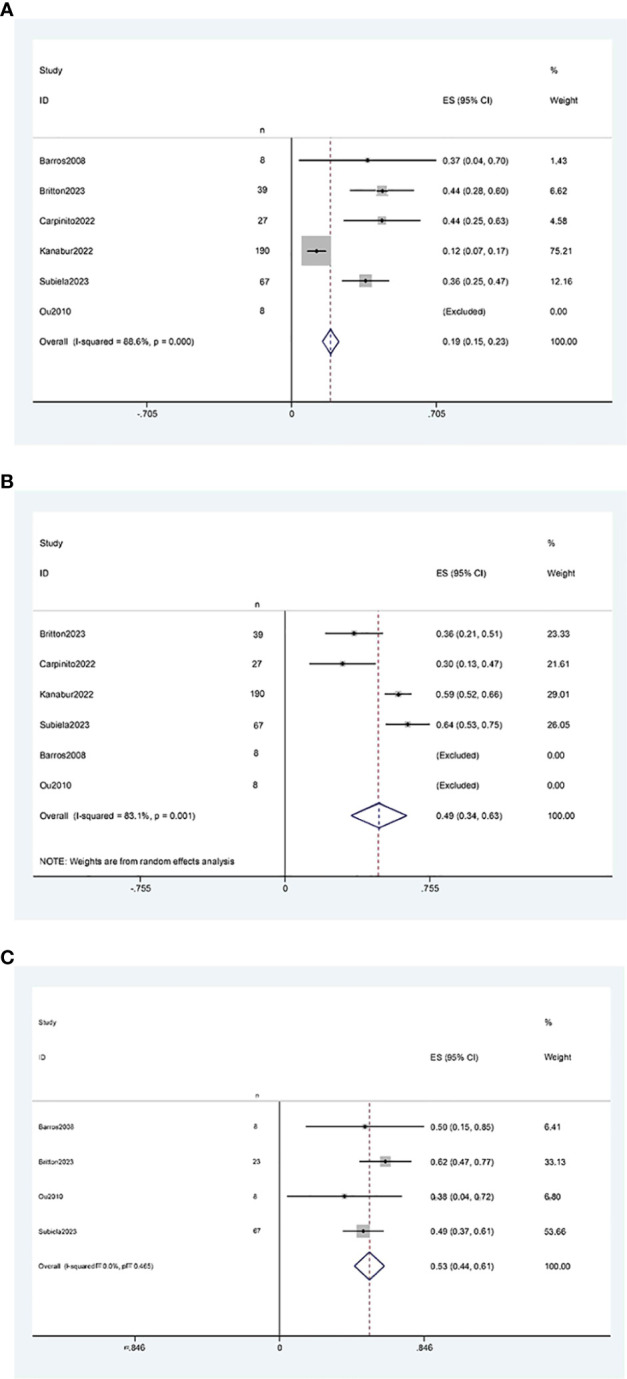
Forest plots of Perioperative outcome: **(A)** minor complications rate was 19% (95% CI 15%, 23%); **(B)** major complications rate was 49% (95% CI 34%, 63%); **(C)** blood transfusion rate was 53% (95% CI 44%, 61%).

**Figure 4:**
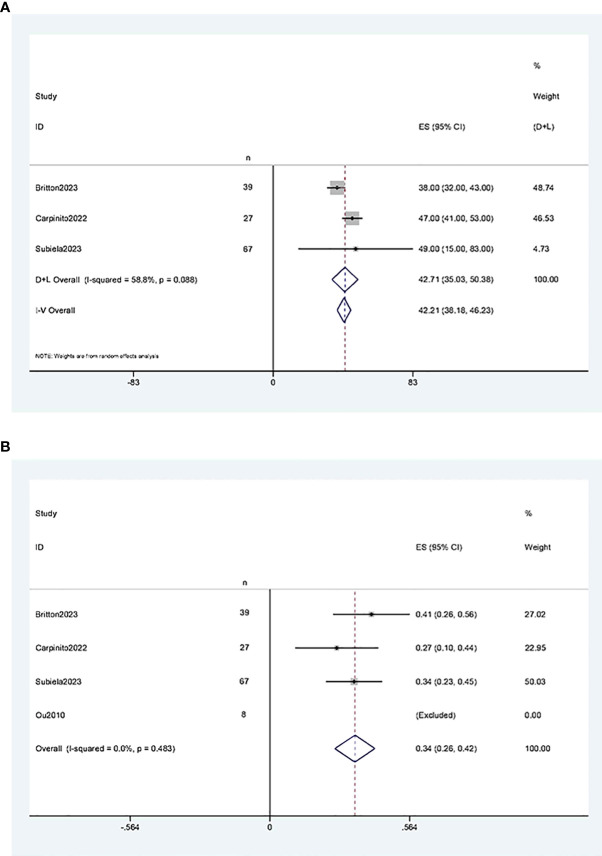
Forest plot**s** of Oncologic Outcomes: **(A)** median survival time was 42.21 months (95% CI 38.18, 46.23); **(B)** tumor metastasis rate was 34% (95% CI 26%, 42%).

The results of the single-arm meta-analysis showed that the operative time for simultaneous radical cystectomy and nephroureterectomy was 378.34 minutes (95% CI 368.95, 387.74, P < 0.001) (**Figure 2A**), the length of hospital stay was 8.04 days (95% CI 7.77, 8.31) (**Figure 2B**), and the blood loss was 711.36 ml (95% CI 638.85, 783.87, P < 0.001) (**Figure 2C**). Additionally, the incidence of postoperative minor complications (Clavien grade 1-2) was 19% (95% CI 15%-23%) (**Figure 3A**), the incidence of postoperative major complications (Clavien grade ≥ 3) was 49% (95% CI 34%-63%) (**Figure 3B**), and the intraoperative blood transfusion rate was 53% (95% CI 44%-61%) (**Figure 3C**). The median survival time was 42.21 months (95% CI 38.18, 46.23) (**Figure 4A**), and the tumor metastasis rate was 34% (95% CI 26%, 42%) (**Figure 4B**).

#### Survival outcome

##### Overall survival

**Figure 5:**
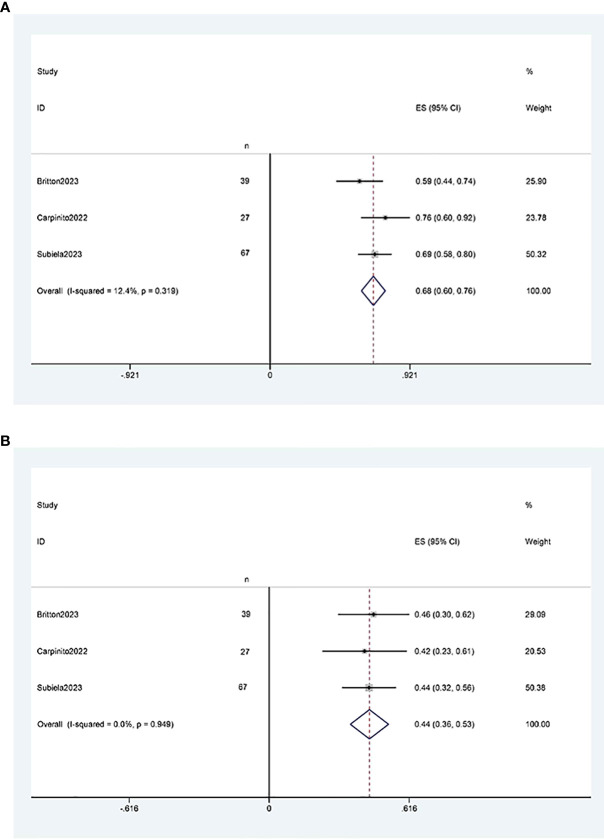
Forest plots of overall survival: **(A)** two years survival rate was 68% (95% CI 60%, 76%); **(B)** five years survival rate was 44% (95% CI 36%, 53%).

Three studies (11, 12, 21) reported two-year and five-year overall survival rates after RCUN. Meta-analysis demonstrated that the overall survival rate was 68% (95% CI 60%, 76%, I^2^ = 12.4%, P < 0.001) at 2 years (**Figure 5A**), and 44% (95% CI 36%, 53%, I^2^ = 0, P < 0.001) at 5 years at 5 years (**Figure 5B**).

##### Recurrence-free survival

**Figure 6:**
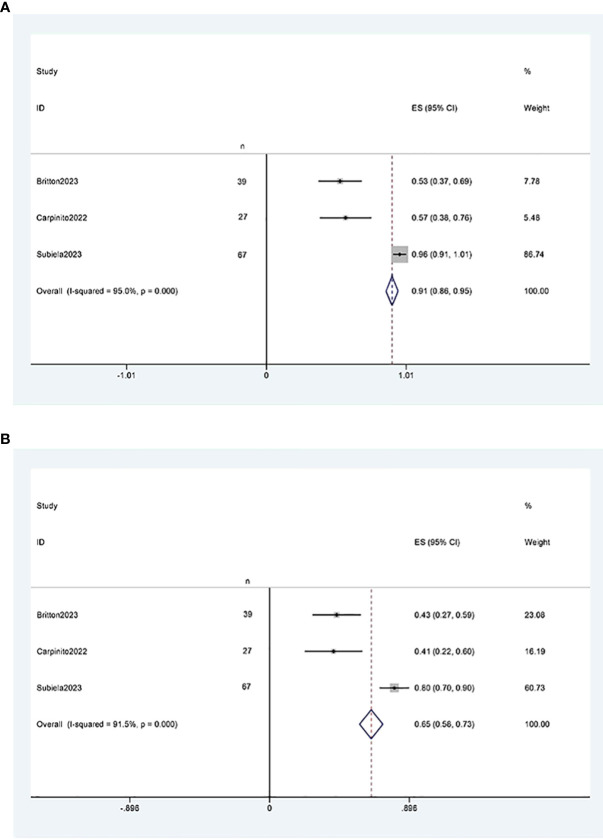
Funnel plot of recurrence-free survival: **(A)** two years survival rate was 91% (95% CI 86%, 95%); **(B)** five years survival rate was 65% (95% CI 58%, 73%).

Three studies (11, 12, 21) reported two-year and five-year recurrence-free survival rates after RCUN. Meta-analysis showed that the recurrence-free survival rate was 91% (95% CI 86%, 95%, I^2^ = 95%, P < 0.001) at 2 years (**Figure 6A**), and 65% (95% CI 58%, 73%, I^2^ = 91.5%, P < 0.001) at 5 years (**Figure 6B**).

##### Cancer-specific survival

**Figure 7:**
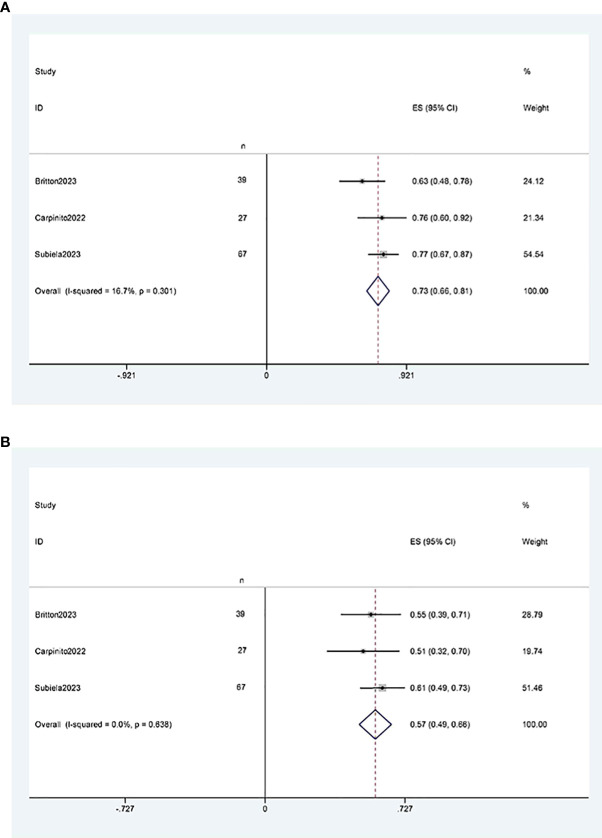
Funnel plot of cancer-specific survival: **(A)** two years survival rate was 73% (95% CI 66%, 81%); **(B)** five years survival rate was 57%(95%CI 49%, 66%).

Three studies (11, 12, 21) reported two-year and five-year cancer-specific survival rates after RCUN. Meta-analysis revealed that the cancer-specific survival rate was 73% (95% CI 66%, 81%, I^2 ^= 16.7%, P < 0.001) at 2 years (**Figure 7A**), and 57%(95%CI 49%, 66%, I^2 ^= 0, P<0.001) at 5 years (**Figure 7B**).

## Discussion

This study represents a systematic review and single-arm meta-analysis investigating the efficacy of simultaneous radical cystectomy and nephroureterectomy (RCUN) in the treatment of Panuc. By conducting a comprehensive evaluation of the relevant literature, we have derived several important conclusions.

First and foremost, our research findings have revealed a disheartening efficacy of RCUN in the treatment of Panuc. Meta-analysis demonstrates a remarkable two-year recurrence-free survival rate exceeding 90%. This outcome can possibly be attributed to the effective eradication of all tumor lesions through simultaneous resection of the bladder and renal ureter, consequently reducing the risks of residual lesions and metastasis. However, the two-year overall survival rate and cancer-specific survival rate both fail to surpass 75%, suggesting that the surgical outcomes may not have met the expected efficacy. It is noteworthy that previous studies have reported a 4-6% urethral recurrence rate in patients undergoing RC (4), and tumor size can nearly directly predict the staging and grading of UTUC (23). Populations from different regions and residing environments may exhibit varying cancer-specific mortality risks in UTUC (24, 25). Furthermore, tumor liver metastasis independently predicts poorer survival rates (26).Risk factors such as smoking, a history of upper tract urothelial carcinoma (UTUC), and chronic kidney disease have also been suggested as potential explanations for malignancy recurrence (27–30). Nonetheless, our survival results indicate that Panuc exhibits a more aggressive behavior compared to primary epithelial UC. Therefore, we propose that RCUN may represent a viable treatment option, particularly for high-risk patients and complex cases involving panurothelial carcinoma.

Secondly, our study also observed a higher risk of surgical complexity and postoperative complications associated with RCUN. This outcome is anticipated as simultaneous excision increases the surgical difficulty and trauma (31–33), potentially resulting in longer operative times and an increased incidence of postoperative complications. Patients diagnosed with preoperative uremia exhibit an elevated risk of intraoperative immune system dysfunction, cardiovascular complications, and anesthesia-related adverse events. Perioperative complications, including bleeding, infection, atelectasis, and cardiovascular or cerebrovascular events, are more likely to occur (34–36). Hence, multidisciplinary collaboration involving anesthesiology, critical care medicine, nephrology, and urology is indispensable for the management of such patients. Consequently, when considering the feasibility of simultaneous surgery, physicians need to carefully evaluate the potential benefits against the surgical risks and the patient’s postoperative rehabilitation requirements. Cutaneous ureterostomy(CU) stands out as a crucial approach for managing early complications, offering advantages such as shorter urinary diversion surgical times, reduced blood loss, lower transfusion rates, and shorter hospital stays (37, 38). When compared to patients undergoing ileal conduit diversion, it may also exhibit a potentially lower occurrence of intraoperative and postoperative complications (39, 40). Current experience with robot-assisted radical cystectomy (RC) patients suggests that there appears to be no significant difference in the incidence of complications between intracorporeal and extracorporeal ileal conduit diversion (41). Given the diverse surgical approaches included in the studies, there is presently an insufficient quantity of data to adequately compare open and minimally invasive treatments for RCUN. However, in the context of radical cystectomy, minimally invasive procedures appear to exhibit a lower transfusion rate in comparison to open surgery (42, 43). Whether this outcome is applicable to RCUN remains subject to verification in subsequent research.

Moreover, given the high risk of recurrent residual urothelium in Panuc (27, 28), regular postoperative testing for residual urothelium should be performed in patients, and prophylactic urethrectomy should even be considered. These findings underscore the importance of meticulous urinary tract management during the perioperative and follow-up periods. Early and long-term follow-up is crucial to timely detect any recurrence. During the perioperative period, patients should receive specialized care and monitoring to ensure proper wound healing. Additionally, appropriate rehabilitation measures, including physical therapy and rehabilitation training, should be implemented to facilitate the recovery of urinary function. These measures should be widely implemented and optimized to provide better treatment options and enhance both survival and quality of life for patients with panurothelial carcinoma.

Furthermore, we recommend comparing our results with alternative treatment modalities such as radiation therapy, chemotherapy, or immunotherapy. Such comparisons will help determine the status and comparative advantages of RCUN in the treatment of panurothelial carcinoma. Unfortunately, the reduced kidney function observed in all RCUN patients in our study makes subsequent treatments, such as chemotherapy, virtually impossible (5, 44, 45). Moreover, integrating study findings with clinical practice guidelines can assist clinicians in formulating more specific treatment plans and decisions tailored to individual patient management.

Finally, we strongly encourage further collaborative and multicenter studies to validate our findings. Multicenter studies can increase sample sizes, enhance the statistical power of the research, and improve the reliability and external validity of the results. Additionally, international collaborative studies can facilitate comparisons between patient populations from diverse regions and ethnicities, enabling a deeper understanding of the effects of RCUN in different demographic groups.

Regarding future research directions, we propose further investigation into the optimal criteria for patient selection in RCUN. While our current study included a diverse range of patients, including high-risk individuals and complex cases, the assessment of feasibility in other patient subgroups was limited. Therefore, future studies should focus on evaluating the efficacy and safety of this surgical approach in specific patient subsets.

Furthermore, an important area of research lies in the technical refinement of simultaneous surgery. The introduction of novel surgical techniques and instruments holds promise for enhancing surgical precision and safety. For instance, the utilization of robot-assisted surgery and microscopy can improve procedural accuracy, visualization, and minimize damage to normal tissue. Comparative studies exploring the effectiveness of different techniques and instruments in simultaneous surgery should be conducted to optimize surgical outcomes (46, 47).

It is important to acknowledge the limitations of our study. The inclusion of a limited number of studies, along with the partial absence of research data and the potential for selection and publication biases, may have influenced our results. Furthermore, our study is based on retrospective data analysis, which only provides a lower level of evidence. It is crucial to carefully weigh the potential benefits against the surgical risks and prioritize postoperative urinary tract management. Future investigations should focus on refining patient selection criteria, technical advancements, and obtaining higher levels of evidence.

## Conclusions

Panurothelial carcinoma represents a rare and highly invasive malignancy. Given the substantial postoperative complications associated with the RCUN approach, a meticulous evaluation of the surgical risks and benefits becomes imperative. Further research and collaborative efforts are warranted to validate our findings, enhance therapeutic strategies, and ultimately ameliorate the prognosis of patients afflicted by upper urinary tract urothelial carcinoma.

## Data availability statement

The original contributions presented in the study are included in the article/supplementary material. Further inquiries can be directed to the corresponding author.

## Author contributions

YL: Protocol development, data collection and management, data analysis and manuscript writing. HZ: Protocol development, data collection and management, data analysis and manuscript writing. XY: Protocol development, data collection and management, data analysis and manuscript writing. ZW: Protocol development, data management, data analysis and manuscript writing. YJ: Data management, data analysis and manuscript writing. JH: Data management, data analysis and manuscript writing. CC: Data management and manuscript writing. CW: Data management and manuscript writing. JW: Data management and manuscript writing. EB: Data management and manuscript writing. All authors contributed to the article and approved the submitted version.
